# Limitations of the particle immunofiltration assay test for diagnosis of heparin‐induced thrombocytopenia

**DOI:** 10.1002/ajh.25901

**Published:** 2020-07-01

**Authors:** Theodore E. Warkentin, Richard J. Cook, Andreas Greinacher

**Affiliations:** ^1^ Department of Pathology and Molecular Medicine Michael G. DeGroote School of Medicine, McMaster University Hamilton Ontario Canada; ^2^ Department of Medicine Michael G. DeGroote School of Medicine, McMaster University Hamilton Ontario Canada; ^3^ Department of Statistics and Actuarial Science University of Waterloo Waterloo Ontario Canada; ^4^ Institut für Immunologie und Transfusionsmedizin, Universitätsmedizin Greifswald Greifswald Germany

To the Editor:

Heparin‐induced thrombocytopenia (HIT) is an immune‐mediated disorder caused by platelet‐activating antibodies that recognize complexes of (cationic) platelet factor 4 (PF4) bound to heparin or certain other polyanions.^1^ Laboratory testing for the pathogenic “HIT antibodies” has focused on platelet activation assays (eg, serotonin‐release assay [SRA]^2^; heparin‐induced platelet activation assay [HIPA])^3^ and PF4‐dependent enzyme‐immunoassays (EIAs).^4^, ^5^ However, assay results are not usually available on the same day of blood draw. Since HIT is ultimately diagnosed in only a minority of patients investigated per clinical suspicion,^6^ and given the need for timely treatment decisions, there is growing interest in rapid immunoassays for HIT.^7^, ^8^ The particle immunofiltration assay [PIFA (HealthTEST Heparin/Platelet Factor 4 Antibody Assay; Akers Biosciences, Inc., Thorofare, NJ]), a rapid immunoassay for detection of PF4/heparin antibodies, received clearance by the U.S. Food and Drug Administration (FDA) in 2004.^9^, ^10^

In 2016, Sun et al^7^ included the PIFA in a systematic review of rapid immunoassays for HIT diagnosis based on one study,^11^ which found 100% PIFA sensitivity, albeit with a wide confidence interval (95% CI, 0.05‐1.00). The wide CI resulted from only two SRA‐positive study patients (as discussed later, these likely were false‐positive SRA results). In contrast, PIFA specificity was only 0.687 (95% CI, 0.586‐0.773). Further, these investigators^7^ were not able to include in their review an earlier 2007 study we reported,^10^ as our results were presented graphically (as ROC curve analyses) without providing the numerical data needed for inclusion in the systematic review. This likely also explains why our joint Hamilton/Greifswald PIFA evaluation (assessing 289 samples, including 25 HIT‐positive patients)^10^ was not included in a later systematic review of rapid immunoassays by Nagler et al.^8^

In the meantime, additional data on the PIFA has become available,^12^, ^13^, ^14^, ^15^ including two studies^13^, ^14^ presented in abstract form at the recent ASH annual meeting (December 2019). We now report the results of our analysis involving the sensitivity and specificity of the PIFA in all English language studies reported to date,^10^, ^11^, ^12^, ^13^, ^14^, ^15^ along with a recent study evaluating a modified PIFA, the PIFA PLUSS.^16^ (The PIFA PLUSS includes a seraSTAT Rapid Blood Cell Separator, allowing for testing of whole blood, rather than serum.^16^) Prompted by a recent report,^17^ we also obtained a report on the results of proficiency testing for the PIFA. Details regarding our systematic review and data synthesis are provided in a supplemental file which includes a PRISMA Flow Diagram (Figure S1 in Appendix S1) and a QUADAS‐2 assessment of study quality (Table S1 in Appendix S1).

We performed three analyses. First, we estimated PIFA sensitivity and specificity for those studies that determined HIT‐positive status by washed platelet activation test (SRA or HIPA) as the reference standard. If the study indicated that a particular sample was positive by SRA or HIPA but negative by PF4‐dependent EIA, the sample was regarded as HIT‐negative. This reduces risk of a false‐positive functional assay result,^9^ and also avoids potential bias towards too negative PIFA assessment because these sera might also not be recognized by other antigen tests. The 95% CIs for the individual studies were computed based on the method of Wilson,^18^ as recommended by Agresti and Coull^19^ for small samples. Overall estimates of PIFA sensitivity and specificity were obtained by jointly synthesizing the data from all seven studies using a bivariate random effects model for meta‐analysis of diagnostic test data, which accommodates study heterogeneity.^20^

Second, for those studies that evaluated samples by both the PIFA and an EIA,^10^, ^11^, ^13^, ^16^ we constructed 2 × 2 tables by cross‐classifying samples according to the two methods. We then assessed the level of agreement between the two assays using Cohen’s kappa statistic along with associated 95% CIs.^21^ An overall measure of agreement was then computed by taking a weighted average of the study‐specific statistics using weights proportional to the inverse of the variances in order to maximize the precision of the resulting estimate.

Third, we obtained the results of a proficiency testing exercise for PIFA which was conducted from 2011 to 2019 by the External Quality Control for Assays and Tests (ECAT) Foundation. In this program, external laboratories tested two samples; one HIT‐positive, the other HIT‐negative. We determined yearly outcomes of participating laboratories obtaining the expected result of positive or negative for the two samples tested.

Figure 1A shows the seven studies (in six reports^10^, ^11^, ^12^, ^13^, ^14^, ^15^) which evaluated the PIFA against a platelet activation reference standard. Test sensitivity ranged from 0.600 to 0.875, except for one study reporting a 0% sensitivity based on 0/15 testing positive; test specificity ranged from 0.311 to 0.895. Combining all studies, the overall estimated sensitivity was 0.665 (95% CI, 0.533‐0.775) and the overall estimated specificity was 0.575 (95% CI, 0.353‐0.771).

**FIGURE 1:**
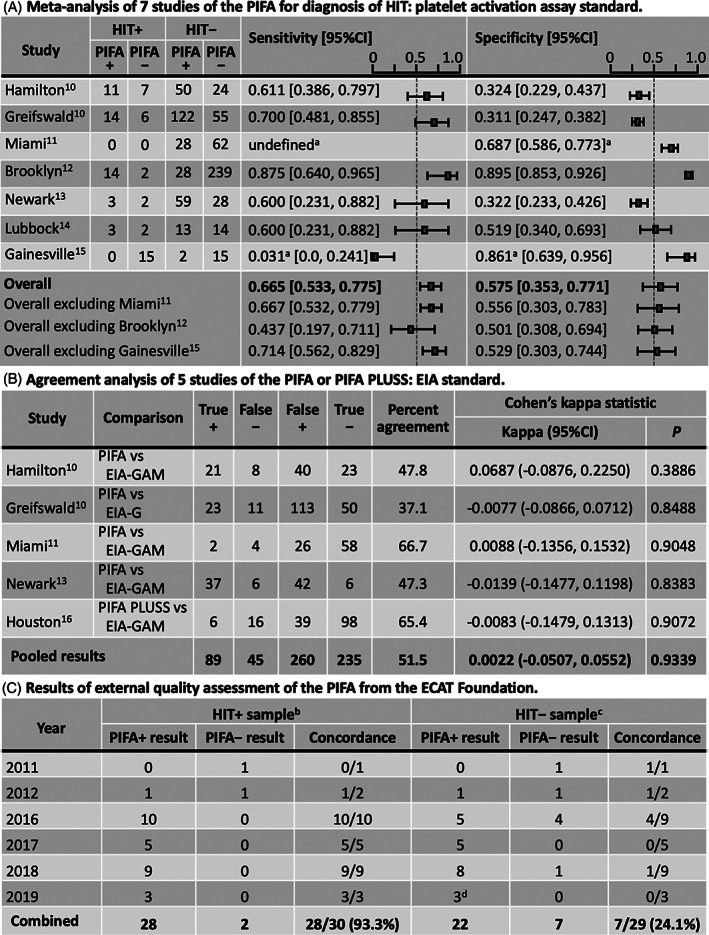
Three evaluations of the PIFA. A, Meta‐analysis of seven studies of the PIFA: platelet activation assay standard. All but one study used the serotonin‐release assay (SRA) as the reference standard; one study (Greifswald) used the heparin‐induced platelet activation (HIPA) test. Abbr.: HIT+, Heparin‐induced thrombocytopenia‐positive; HIT−, HIT‐negative; PIFA+, Particle ImmunoFiltration Assay‐positive; PIFA−, PIFA‐negative. The LR+ and LR− calculations, based upon the overall meta‐analysis estimates of sensitivity and specificity (0.665 and 0.575, respectively), are as follows: (a) LR+ = sensitivity/[1‐specificity] = 0.665/[1‐0.575] = 1.56. (b) LR− = [1‐sensitivity]/specificity = [1‐0.665]/0.575 = 0.583. B, Agreement analysis of five studies of the PIFA or PIFA PLUSS: EIA standard. Abbr.: +, positive; −, negative; EIA‐G, IgG‐specific enzyme‐immunoassay; EIA‐GAM, polyspecific enzyme‐immunoassay that detects antibodies of IgG, IgA, and/or IgM classes. C, Results of external quality assessment of the PIFA from the ECAT Foundation. No data were available for 2013, 2014, or 2015. During each of the six years shown, each participating laboratory received one HIT‐positive and one HIT‐negative sample. ^a^ For performing the meta‐analysis, a continuity correction of 0.5 was added to all cells for the Miami and Gainesville studies (because one or more cells for these studies had a value of 0). ^b^HIT‐positive samples were prepared by diluting a strong‐positive HIT sample with normal pooled plasma. ^c^HIT‐negative samples were normal pooled plasma. ^d^One borderline positive sample was classified as “positive” for purposes of analysis

In our evaluation of the Miami study,^11^ both SRA‐positive patients were classified as HIT‐negative based upon negative EIA results (these patients also had low 4Ts scores and were not regarded by the study authors as having had HIT).^11^ We therefore also assessed overall PIFA sensitivity and specificity omitting the Miami study (as there were no HIT‐positive subjects to judge test sensitivity). We performed another analysis omitting the Brooklyn study (which was reported in abstract form in 2014 and did not give a comparison with an EIA).^12^ We also performed an additional analysis omitting the Gainesville study (as this study appeared to be an outlier).^15^ All estimated sensitivities were below 0.714, corresponding to values too low for an acceptable screening test; further, no analysis showed an estimated specificity greater than 0.575.

Figure 1B shows those studies^10^, ^11^, ^13^, ^16^ that permit comparison of PIFA reactivity vs an EIA. None of the five studies yielded CIs demonstrating improvement over chance agreement. Moreover, when pooling the kappa statistic across studies, the overall measure did not suggest agreement beyond chance. Indeed, the overall raw agreement (pooled data) showed only 51.5% agreement. These results contrast with data presented on two FDA websites,^22^, ^23^ suggesting assay performance may have changed.

Figure 1C shows the results of the ECAT Foundation external quality assessment. The external laboratories generally obtained a positive PIFA result for the six HIT‐positive samples evaluated (28/30 [93.3%]); however, the laboratories also tended to obtain a positive PIFA result for the corresponding 6 HIT‐*negative* samples, that is, the expected negative results were seen in only 7/29 (24.1%) of the HIT‐negative samples.

We note that poor assay performance can cause problems in patient management, as illustrated by a report^24^ of a patient with a clinical picture of HIT (thrombocytopenia; necrotizing skin lesions at heparin injection sites; deep‐vein thrombosis; post‐heparin bolus anaphylactoid reaction) and strong‐positive testing by EIA and SRA; however, the PIFA test was repeatedly negative.

One way of assessing assay utility is through evaluation of its impact on clinical decision making. For example, the likelihood ratio of a positive test result (LR+) and the likelihood ratio of a negative test result (LR−) reflect how the odds of disease are altered with a positive and negative test result, respectively. For the PIFA, the overall LR+ (sensitivity/[1‐specificity]) and LR− ([1‐sensitivity]/specificity) are 1.56 and 0.582, respectively (see Figure legend for detailed calculations); thus, for a patient judged clinically to have a 50% probability of HIT, the pre‐test odds are 1 (0.50/0.50), and the resulting post‐test probabilities are approximately 0.60 and 0.37 for a positive and negative PIFA test result, respectively; these values are not so different from the initial starting estimate (0.50). In contrast, the LR+ and LR− values for the EIA (~6 and ~0.01, respectively)^25^ would result in post‐test probabilities of 0.86 and <0.01, and for two other rapid assays^25^, ^26^ the corresponding LR+ values (~16 and ~66, respectively) and LR− values (approximately 0.034 and 0.031, respectively) would result in post‐test probabilities of 0.94‐0.98 and 0.03, respectively. Moreover, whereas the PIFA only provides a binary outcome (positive/negative), the EIA and other rapid immunoassays provide semiquantitative results, allowing for even greater estimates of LR+ for strong‐positive results.^25^, ^26^

The poor performance of the PIFA is clear from Figure 1A,B. Notably, there is significant heterogeneity between the studies with homogeneity tests yielding *P* < .001 for both sensitivity and specificity. Also notable, however, is the strong consistency in the results among the participating laboratories from the ECAT Foundation program, with results consistently incorrect for most of the HIT‐negative samples.

In summary, our analysis of available data indicates that the PIFA provides minimal if any value for HIT diagnosis. Further, PIFA results do not correlate with EIA reactivity. Overall, the data are compatible with a test that yields a positive result approximately 42% of the time (all PIFA studies pooled^10^, ^11^, ^12^, ^13^, ^14^, ^15^), with minimal if any association with whether the patient has HIT or not (Figure 1A), or indeed whether anti‐PF4/heparin antibodies detectable by EIA are present or not (Figure 1B). As indicated by the single PIFA PLUSS study,^16^ the new test version has similar suboptimal performance. The ECAT Foundation proficiency testing evaluation also raises concerns on the ability of the PIFA to distinguish between positive and negative HIT samples. Pending future supportive data, the PIFA test bears substantial risk for HIT overdiagnosis and, in our opinion, also for false‐negative results pointing away from a true diagnosis of HIT.

## CONFLICT OF INTEREST

T.E.W. has received lecture honoraria from Alexion and Instrumentation Laboratory and royalties from Informa (Taylor & Francis); has provided consulting services to Aspen Global, Bayer, CSL Behring, Ergomed, Instrumentation Laboratory, and Octapharma; has received research funding from Instrumentation Laboratory; and has provided expert witness testimony relating to heparin induced thrombocytopenia (HIT) and non HIT thrombocytopenic and coagulopathic disorders, including on utility of laboratory assays for HIT. A.G. has received lecture honoraria from Instrumentation Laboratory and royalties from Informa (Taylor & Francis); has provided consulting services to Aspen Global, Aspen Germany, Ergomed, and Chromatec; and has received research funding from BioKit. R.J.C. discloses no relevant conflicts of interest.

ORCID profiles: T.E.W. 0000‐0002‐8046‐7588; A.G. 0000‐0001‐8343‐7336.

## AUTHOR CONTRIBUTIONS

T.E.W. designed the study, reviewed the Hamilton data, analyzed the publically available PIFA data, and wrote the first draft of the manuscript. A.G. reviewed the Greifswald data and helped edit the manuscript. R.J.C. provided statistical expertise and analyses. All three authors approved the final version of the paper.

## Supporting information


**Appendix** **S1.** Supporting Information.
