# Supplementary material for: Limitations of the particle immunofiltration assay test for diagnosis of heparin‐induced thrombocytopenia
Source: Am J Hematol. 2020 Jul 1;95(9):E250–4. doi: 10.1002/ajh.25901 (PMC7496181; doi:10.1002/ajh.25901)
Supplement: Supplementary file 1 — Appendix S1. Supporting Information. [file AJH-95-E250-s001.docx]

**PRISMA Flow Diagram for PIFA Meta-Analysis**

Identification

Duplicate records excluded
(N=22)

Total records identified

(N=46)

Additional records identified through other sources
(N=3)

*Bibliography review (N=2)*

*Other (N=1)*

Records identified through database searching (N=43)

*PubMed (N=9)*

*EMBASE (N=30)*

*COCHRANE (N=4)*

Screening

Eligibility

Records excluded by title and abstract review
(N=15)

Records screened after duplicates removed
(N=24)

Full-text articles/abstracts excluded (N=2)

- *No laboratory test reference standard (SRA/HIPA, LIA); article in Chinese (N=1^a^)*
- *Unable to construct 2x2 table against laboratory test reference (N=1^b^)*

Full-text articles/abstracts assessed for eligibility
(N=9)

Included

Studies included in qualitative synthesis

(N=8^c^)

Figure 1. PRIMSA flow diagram for the selection, assessment and exclusion of studies.

The literature search was performed using keywords: “PIFA” and “heparin”; and “particle immunofiltration” and “heparin.”

Footnotes for the PRISMA flow diagram:

^a^ Li S, Fan LK, Wu W, Zhao YQ, Wang SJ. [Diagnostic value of two immunoassays for detecting heparin/PF4 complex antibodies in heparin-induced thrombocytopenia] *Chin J Hematol*. 2019; 40 (5): 411-416. [article in Chinese]

^b^ Francis JL, Drexler A, Duncan MK, Desai H, Amaya M, Robson T, Meyer TV, Reyes E, Rathmann K, Amirkhosravi A. Prospective evaluation of laboratory tests for the diagnosis of heparin-induced thrombocytopenia. *Blood*. 2006; 108: 312a [Abstr].

^c^ One full-length article described two studies, one from Hamilton (Canada) and the other from Greifswald (Germany).

Supplemental Table 1. Study quality assessment by QUADAS-2 criteria.

|  | Risk of bias | | | | Applicability concerns | | |
| --- | --- | --- | --- | --- | --- | --- | --- |
| Studies | Patient selection | Index test | Reference standard | Flow and timing | Patient selection | Index test | Reference standard |
| Eight studies included in quantitative analysis | | | | | | | |
| Hamilton^10^ | Low | Low | Low | Low | Low | Low | Low |
| Greifswald^10^ | Low | Low | Low | Low | Low | Low | Low |
| Miami^11^ | Low | Low | High^a^ | Low | Low | Low | High^a^ |
| Brooklyn^12^ | Low | Low | High^b^ | Low | Low | Low | High^b^ |
| Newark^13^ | High^c^ | Low | Low | High^c^ | High^c^ | Low | Low |
| Lubbock^14^ | High^d^ | Low | Unclear^e^ | High^d^ | High^d^ | Low | Unclear^e^ |
| Gainesville^15^ | Unclear^f^ | Low | Low | High^f^ | Unclear^f^ | Low | Low |
| Houston^16^ | Low | Low | Low^g^ | High^g^ | Low | Low | Low^g^ |

Note that 4/8 studies listed (Brooklyn, Newark, Lubbock, Gainesville) were reported only as abstracts (Brooklyn in 2014, the others in 2019).

References are shown on the following page (reference numbering per main paper).

^a^ All SRA-positive samples were EIA-negative, raising concerns about SRA performance.

^b^ No EIA testing performed, so false-positive SRA results cannot be ruled out.

^c^ Algorithm included automatic reflexing of PIFA-positive samples for further evaluation by SRA and EIA, whereas evaluation of PIFA-negative samples was at discretion of clinicians.

^d^ only 32/170 patients evaluated by PIFA underwent testing by the reference standard SRA.

^e^ EIAs were not performed, so quality check against false-positive SRA results not available.

^f^ Uncertain how samples for HIT testing were identified and criteria for referral for reference testing (SRA) not stated (only 32/60 EIA-positive samples were referred for SRA testing); accordingly this study was not included in the final quantitative analysis.

^g^ All samples underwent testing by PIFA and polyspecific EIA; however, only PIFA-negative samples were potentially referred for SRA testing, and so an assessment of PIFA performance against HIT-positive/HIT-negative status by SRA was not feasible.

References for supplemental table 1 (same reference numbering as per main paper).

10. Warkentin TE, Sheppard JI, Raschke R, Greinacher A. Performance characteristics of a rapid assay for anti-PF4/heparin antibodies, the Particle ImmunoFiltration Assay. *J Thromb Haemost*. 2007;5(11):2308-2310.

11. Andrews DM, Cubillos GF, Paulino SK, Seckinger DL, Kett DH. Prospective observational evaluation of the particle immunofiltration anti-platelet factor 4 rapid assay in MICU patients with thrombocytopenia. *Crit Care*. 2013;17 (4):R143.

12. Nannapaneni S, Malhotra I, Simon M, et al. A rapid heparin antibody detection assay performs better than 4T’s score in predicting HIT diagnosis: a single community medical center retrospective analysis [abstract]. *Blood*. 2014;124(21):1457.

13. Kra J, Horng H. Utility and pitfalls of stepwise laboratory testing for heparin induced thrombocytopenia: retrospective review from an academic medical center [abstract]. *Blood*. 2019;132(Suppl. 1):2371.

14. Ball S, Adhikari N, Sultan A, et al. Effective implementation of a structured protocol for facilitation of the judicious use of antibody test for diagnosis of heparin induced thrombocytopenia [abstract]. *Blood*. 2019;134(Suppl. 1):3462.

15. De Luna M. HIT: Anti-platelet factor 4-heparin (pf4-h) assay validation and comparison study [abstract]. *Am J Clin Path*. 2019; 152 (Suppl. 1): S124.

16. Compton FB, Alrabeh R, Nguyen LQ, Nedel cu E, Wahed A, Nguyen ND. PIFA PLUSS P4 assay for screening of heparin-induced thrombocytopenia. *Lab Med*. 2019;50(1):73-77.
